# Effect of Whole-Body Vibration on Serum Levels of Brain Derived Neurotrophic Factor and Cortisol in Young, Healthy Women

**DOI:** 10.3390/ijerph192316108

**Published:** 2022-12-01

**Authors:** Anna Piotrowska, Halina Gattner, Justyna Adamiak, Sylwia Mętel, Olga Czerwińska-Ledwig, Wanda Pilch, Ewa Sadowska-Krępa, Małgorzata Żychowska, Ivan Uher, Tomasz Pałka

**Affiliations:** 1 Department of Chemistry and Biochemistry, Faculty of Physiotherapy, University of Physical Education in Kraków, Jana Pawła II Avenue 78, 31-571 Krakow, Poland; 2 Faculty of Physiotherapy, University of Physical Education in Kraków, Jana Pawła II Avenue 78, 31-571 Krakow, Poland; 3 Institute of Applied Sciences, Faculty of Physiotherapy, University of Physical Education in Kraków, Jana Pawła II Avenue 78, 31-571 Krakow, Poland; 4 Institute of Sport Sciences, Department of Physiological and Medical Sciences, The Jerzy Kukuczka Academy of Physical Education in Katowice, Mikołowska Street 72a, 40-065 Katowice, Poland; 5 Department of Sport, Faculty of Physical Education, Kazimierz Wielki University in Bydgoszcz, Chodkiewicza Street 30, 85-091 Bydgoszcz, Poland; 6 Institute of Physical Activity and Sports, Pavol Jozef Šafárik University, 04001 Košice, Slovakia; 7 Department of Physiology and Biochemistry, Faculty of Physical Education and Sport, University of Physical Education in Kraków, Jana Pawła II Avenue 78, 31-571 Krakow, Poland

**Keywords:** whole body vibration, BDNF, cortisol, vibration, exercise

## Abstract

Vibration exercises on a platform (whole-body vibration, WBV), widely used in rehabilitation, sports medicine, and fitness, is an alternative to strength effort. The presented study assessed the effect of a 12-week cycle of vibration training on the serum concentrations of brain-derived neurotrophic factor (BDNF) and cortisol in young women (trial ID: ACTRN 12621000114842). Volunteers were assigned to three groups: performing exercises on a vibrating platform (n = 17), performing identical exercises without a platform (n = 12), and passive control group (n = 17). The concentration of BDNF and cortisol was assessed four times: before the first training session, 5 min after it, also before, and 5 min after the last training session. There were no statistically significant changes in the groups or among groups for both substances. WBV in the presented form did not increase the secretion of BDNF and is not a stressful stimulus.

## 1. Introduction

Whole-body vibration (WBV) is used in rehabilitation, sports medicine, and fitness and is an alternative to traditional strength effort. The essence is mechanical vibrations generated by a vibrating platform and transferred deep into the body through the myofascial and skeletal systems of the person subjected to vibration. The biomechanical characteristics of the resulting sinusoidal oscillations are frequency, amplitude, and acceleration. During WBV, static exercises (holding a given position) or dynamic exercises (combined with limb movement) can be performed [1,2]. 

The mechanism of the beneficial effects of WBV is combined with the tonic vibration reflex in which the activation of mono- and polysynaptic neural pathways in response to vibration of a given intensity is observed. In the next stage, there is efferent impulsion from the spinal cord and the contraction of muscle cells. The tonic vibration reflex active during WBV generates alternating muscle contractions and relaxation. Mechanical vibrations also generate remodeling in the skeletal system [3]. The influence of WBV on the human endocrine system has also been documented [4,5]. 

On the other hand, this indicates that improperly applied vibrations may distress the human body. The first known type of such action was vibration syndrome [6]. It is now known that not only vibration white finger can be a consequence of exposure to vibrations. Body vibration is increasingly identified as a source of back pain [7]. Occupational exposure to vibration is associated with neck pain [8,9]. The mechanism of this dependence is still unclear, but programs aim to eliminate the health consequences of various occupational groups [9,10]. 

There are considerable differences in the regional amounts of the brain-derived neurotrophic factor (BDNF) protein. Its expression also takes place outside the central nervous system. BDNF belongs to the neurotrophin family and increases neuronal survival and neuroplasticity. In a healthy population, the concentration of this factor may be highly varied [11]. However, in many neurodegenerative and neuropsychiatric disorders (e.g., depression [12], bipolar disorder [13] or schizophrenia [14], and neurodegenerative diseases [15]), it is known to be reduced. For this reason, methods are being sought to stimulate increasing the plasma level of BDNF, which can aid in reversing or stopping the progression of psychiatric or neurodegenerative diseases. It is currently known that factors increasing BDNF expression include pharmacological therapy with antidepressants [16] and physical activity [15,17]. However, the type, intensity, duration, and frequency of physical activity differ in their effect on BDNF levels. A single sustained strength effort has little or no effect on the basal level of this neurotrophin. In contrast, a single, exceptionally long-lasting, and repeated aerobic exercise may increase circulating BDNF [18].

The cortisol release is associated with psychological and physical stress [19]. There are pieces of evidence that stress-induced disruption of the circadian rhythm of cortisol secretion has negative consequences for brain health. It is frequently investigated as a biomarker of stress and a potential intermediary between stress and impaired brain function [19,20]. Cortisol is a powerful hormone that affects cognition in multiple and complex ways. Associations between the circadian rhythm of cortisol secretion and both memories and executive functions are shown with its potential role as a neuroendocrine 24-h interval signal that synchronizes peripheral clocks throughout the brain to enable optimum function [21]. Exercise represents physical stress, which challenges homeostasis [22]. In response to this stressor, the autonomic nervous system and the hypothalamic–pituitary–adrenal axis are known to react and participate in the maintenance of homeostasis. This includes the elevation of cortisol in plasma. During training, adaptative changes lead to the normalization of hormone levels. Exercise is now recognized as a simple tool to relieve mental stress. Regular physical activity counteracts the effects of sudden and chronic stress, ensures better tolerance of stress loads, and has a positive effect on reducing the risk of diseases related to the presence of chronic stress and chronic hypercortisolemia [23].

WBV, due to the activation of the tonic vibration reflex [24] and other mechanisms, can be considered as a substitute for physical activity. It was shown that not for general, but for some, selected populations, WBV can be used as an initial form of exercise or exercise adjuvant [1]. However, in most cases, the effectiveness was only studied in men [1]. The influence of female hormones may modulate the training effects [25,26]. The study analyzed changes in the levels of BDNF as a neurotrophic factor involved in the maintenance and improvement of cognitive functions and cortisol as a stress marker in the blood of young, healthy women after a single session and after 12 weeks of exercise combined with WBV.

The presented hypothesis assumed that the proposed training with an additional vibration stimulus would substantially impact the plasma level of BDNF than training without a vibration platform. A 12-week vibration training series can increase this neurotrophy’s concentration, which can be seen by comparing the levels before the first and the last training. The first training session can be a stress factor, which can be illustrated by an increase in the plasma cortisol concentration of the subjects. Regular training, however, should have an adaptive effect. It was expected that the last training session would no longer increase the cortisol levels. Regular exercise could also lower the steady-state levels of this hormone.

## 2. Materials and Methods

### 2.1. Participants

Young, untrained women (n = 50) who met the following inclusion criteria were qualified to participate in the study. The following inclusion criteria were used: age from 18 to 25 years, body mass index (BMI) within the range of expected values (18.5–24.9), diet consistent with the recommendations of the Institute of Food and Nutrition [27], a level of physical activity determined based on the International Physical Activity Questionnaire (IPAQ) [28] as low (insufficient). The exclusion criteria were multiple medical burdens confirmed by medical qualification that constitute a contraindication to WBV, as indicated in Table 1. 

Participants were informed about the purpose, methods used, and the possibility of withdrawing from the study at any time. The subjects gave their written consent and agreed to stick to the current diet, not to take any medications or alcohol, and to follow the current level of physical activity throughout their participation in the program. 

Allocation of subjects into different groups was completed through volunteer sampling—the participants were recruited via posted advertisement at the university and assigned to the following groups: the experimental group (EG) performing exercises on a vibrating platform; the active control group (AC) performing identical exercises without a platform; and the passive control group (CG) not doing any exercises. 

**Figure 1:**
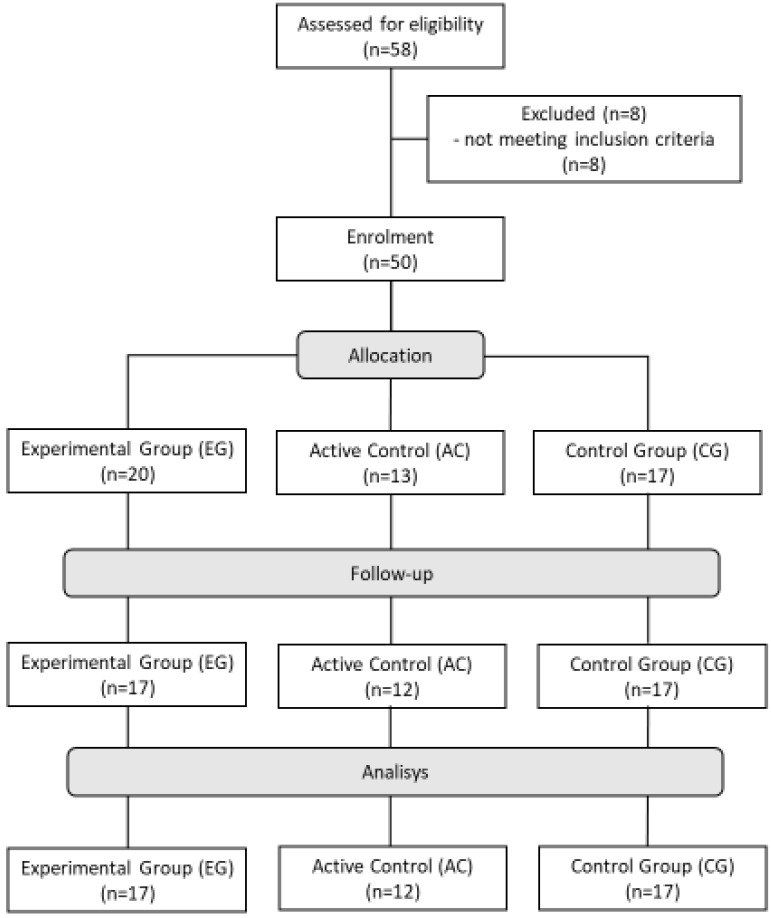
Patient flow diagram (www.consort-statement.org, accessed on 16 October 2022).

During the project, eight women were excluded (Figure 1). Women who were excluded from the study were participants who did not complete the assumed amount of training. This was due to upper respiratory tract infections and the need to start antibiotic therapy (one participant) or to give up due to other private reasons. The age and essential anthropometric characteristics of the studied women are shown in Table 2.

### 2.2. Methods

#### 2.2.1. Anthropometric Measurements

Measurements of weight and body composition were performed twice with the TANITA BC 418 MA (93/42 EEC) (Tanita, Tokyo, Japan) body composition analyzer following the guidelines of Ackland et al. [30] before and after the end of the 12-week WBV. The participants’ height was measured using an anthropometer, and the BMI index was calculated according to the generally accepted formula.

#### 2.2.2. Blood Collection Method and Biochemical Determinations

Venous blood was collected four times (I—prior to the first training session, II—up to 5 min after the first training session, III—prior to the last training session, IV—up to 5 min after the last session). Blood samples were collected in the morning (7.00–9.00) considering the daily fluctuations with the use of a BD Vacutainer system (Becton Dickinson, Franklin Lakes, NJ, USA). Given the menstruation cycle, the study was designed so that all participants were in the same phase of the cycle (follicular phase of the menstrual cycle) at the time of blood collection.

In the obtained serum, the concentrations of human brain-derived neurotrophic factor (BDNF) (ELISA Kit 201-12-1303, SRB/Shanghai) and cortisol (cortisol, human EIA1887, DRG, Marburg, Germany) were determined using the enzyme immunoassay.

#### 2.2.3. Whole-Body Vibration

The Fitvibe Excel Pro vibrating platform (Gymna Uniphy, Bilzen, Belgium), generating vertical vibrations (amplitude: 2 and 4 mm, frequency 20–60 Hz), was the source of the vibration stimulus. The examined women participated in individual, supervised sessions three times a week for three months. On the platform, in a standing position with slightly bent knees (15%), the participants received vibrations transmitted symmetrically to both sides of the body. To verify the transmission of vibrations to the head and neck of the participants and to monitor the training intensity (talk test), they were asked to maintain verbal contact and the body position was also corrected each time by the instructor.

**Figure 2:**
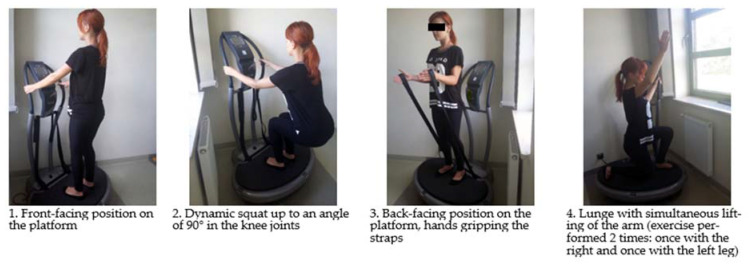
Exercises on the vibrating platform performed in the main part of the training session.

Each session included exercises in one series performed in antigravity positions on a vibrating platform (Figure 2). Dynamic exercises were performed to the rhythm of a metronome—25 movements per minute. The women marched around the room during the break between the main exercises. Every training session began with a 5-min warm-up in a standing position and ended with a 5-min relaxation exercise in low positions on a mattress. In order to strengthen the antigravity control and reduce the transmission of vibrations to the head, the examined women performed exercises in positions corrected by the instructor in order to maintain the alignment of the limb joints with slightly bent knee joints (15%) and raised heels, and the linearity of the body diaphragm system (breathing pattern optimization).

Great emphasis was placed on the individual control of each participant while exercising on the vibrating platform. Attention was paid to the correct body posture. This was to limit the transmission of vibrations to the head and neck. During the entire investigation, no withdrawal due to headaches, excessive tension in the muscles, or other disturbing symptoms through training allowed for the presumption that maintaining the correct body position while performing exercise on the vibration platform ensured the safety of the participants. 

Training Schedule

- 1–4 weeks: Duration of each exercise—1 min; break time between exercises—1 min; vibration parameters—frequency 40 Hz, amplitude 2 mm;
- 5–8 weeks: Duration of each exercise—1 min; break time between exercises—1 min; vibration parameters—frequency 45 Hz, amplitude 2 mm;
- 9–12 weeks: Duration of each exercise—1 min; break time between exercises—1 min; vibration parameters—frequency 50 Hz, amplitude 2 mm.

#### 2.2.4. Ethics Approval and Consent to Participate

The project was approved by the Ethics Committee (consent number: 224/KBL/OIL/2016) and registered in the clinical trial database (trial ID: ACTRN 12621000114842). 

#### 2.2.5. Statistical Analysis

Statistical analyses were conducted using STATISTICA 13.1 (StatSoft, Poland). The results are presented as the mean, standard deviation (SD), median (ME), minimum value (MIN), and maximum value (MAX). The normality was tested with the Shapiro–Wilk and the homogeneity of variance with the Levene tests. Outliers were rejected based on the Grubbs test. The results within one group were compared with one-way ANOVA for repeated measures. The results obtained for the three selected groups were compared with one-way ANOVA for independent measures with Tukey’s post hoc test for unequal counts. The FFM was added as a covariable in order to assess it as a factor differentiating the results between the study groups (ANCOVA model). The level of significance (α) was set at *p* < 0.05.

## 3. Results

As a result of the conducted analyses, statistically significant intergroup differences in body weight (*p* = 0.029) and fat-free mass (*p* = 0.013) were found. Women from the EG group were characterized by a lower fat mass and fat-free mass compared to those within the CG. There were no significant differences between group membership regarding the variables: age, height, percentage of body fat, and BMI (Table 2).

The analysis of the serum cortisol level results showed that the selected groups did not differ in the initial concentration of this hormone. In the groups performing exercises (EG and AC), the effect of one-time training on the cortisol levels was not indicated, regardless of whether the exercises were performed with or without the use of a vibrating platform. There was also no indication of a change after three months of exercise. A comparison of the plasma cortisol concentrations measured before and after the last training also did not show significant differences, with the last approximately the same as the first session (Table 3).

When analyzing the concentration of BDNF and cortisol in the plasma, similar values were observed (Table 4). Baseline levels of this neurotrophin did not differ between the selected groups. It also remained the same after the first training in the groups performing exercises on the platform (EG) and without the platform (AC). BDNF concentrations in the plasma of the test persons did not differ before and after the last training session. The influence of a 12-week series of WBV exercises on the level of this factor was not indicated.

Significant differences in FFM between the groups indicated the need to analyze the FFM as a variable influencing the concentration of the studied criteria. ANCOVA, for the results obtained before the first training for all selected groups, demonstrated that FFM did not influence the level of BDNF (*p* = 0.481) or cortisol (*p* = 0.241). This analysis was repeated for the third measurement (before the last vibration training) with similar results (*p* = 0.752 and *p* = 0.311, respectively).

## 4. Discussion

This study shows that both the one-time and three-month period with or without the addition of a vibration stimulus does not affect the concentration of cortisol and BDNF in young, healthy women. It was observed that exercises on a vibrating platform at low training loads in the indicated population did not significantly affect the profile of the examined factors. The study demonstrated that WBV exercises, when performed individually, according to the instructor’s guidelines, constitute a well- accepted and not overly stressful form of physical activity for young women. In the course of this study, the authors followed the methodology postulated by Wuestefeld et al. as much as possible [31]. 

Despite the sizeable individual diversity of somatic parameters, three groups of women were recruited that did not differ in the initial BDNF level. An important finding was also the circadian rhythm of BDNF release, hence the need to perform exercise tests at a fixed time (preferably between 7.00 and 9.00 am) and fluctuations in the level of this neurotrophin associated with the monthly cycle in women, mainly imitating the profile of sex steroid concentrations [32]. Strict adherence to the research protocol allowed us to obtain reliable results. It was essential to adjust the beginning of the training cycle for each woman to begin and end in her follicular phase of the monthly cycle. It is claimed that engaging in various physical pursuits increases neuronal activity [17]. Prolonged physical exertion may increase neuroplasticity and initiate processes leading to improving or maintaining cognitive functions. This effect has been proven in clinical trials in healthy volunteers [17] and patients with symptoms of cognitive dysfunction [33]. More recent studies have shown that women subjected to a combination of physical training and vibration had higher plasma levels of BDNF. However, the average age of those participants was higher than in the presented study; moreover, they exhibited health burdens [34,35]. Based on the bring forward observation, the 3-month WBV exercises did not provide a sufficient load to exhibit a profound effect in young, healthy women.

On the other hand, the physical effort performed by the women on the vibrating platform as well as the action of the vibrations themselves were not a stress factor for the subjects, which is also important for the recovery of a good postural control pattern and the breathing quality of the exercising women. This was evidenced by the lack of changes in cortisol concentration after a single WBV and the entire program. The cortisol concentration modulates the circadian rhythm. Its highest concentration in the blood was observed, as in the case of BDNF in the morning hours. As a result, the physiological range of concentrations in the morning at rest for cortisol was 150–650 nmol L^−1^. This study’s results were within the physiological reference ranges before and after the training session. 

The intensity or duration of exercise determines the physical stress and the response of the adrenal cortex. Post-exercise maintenance of cortisol secretion depends on the degree of training, environmental conditions, pre-exercise glycemia, and other variables. 

The assessment of the effect of WBV on the level of selected hormones including cortisol was investigated by Kvorning et al. [36]. The cortisol levels increased and decreased after the vibratory stimulus was applied during the first training session, but did not change following nine weeks of WBV. The introduced investigation observed only a slight drift toward a post-training spike in the cortisol levels after the first training session.

In contrast to set forth observations, Kvorning [36] observed higher cortisol levels after the completion of the program. These differences seem to result from different training protocols, as indicated by Erskine et al. [4], where changes in the cortisol concentration (assessed in saliva) did not alter after the completion of isometric exercise training. In addition, Erskine et al. [4] found no change in the salivary testosterone or cortisol levels immediately post-, one h post-, two h post-, and 24 h post-vibration; however, there was a tendency (*p* = 0.052) for cortisol levels to rise across time in the vibrated group. Nevertheless, the authors concluded that the observed differences in cortisol levels for young, healthy individuals did not stimulate the neuroendocrine system in a significant way. However, some differences could be observed in the elderly. Hormonal fluctuations were noted in elderly individuals following a single session of WBV. Immediately after the WBV session, the cortisol levels were higher than those observed with static squats alone. Still, by 1 h and 2 h-post-treatment, the cortisol concentrations were reduced below the pre-treatment levels with both WBV plus static squat and static squat alone [29].

Despite the gradual increase in the intensity of the vibration stimulus (change of frequency in individual weeks of using exercises on the platform), no significant outcomes were observed. The gradation of the intensity of exercises on the platform should also be applied to obtain beneficial effects. 

### 4.1. Study Limitations

This study only included women with well-defined age and baseline physical activity characteristics that cannot be directly interpolated into the general population. The investigation was solely concerned with the proposed training cycle with specific vibration parameters used in the following weeks of conducting physical exercises on a vibration platform. The change in the device generating the vibration buffer and the modification of the frequency and amplitude may influence the analyzed markers. A significant limitation was related to the size of the cohort group.

### 4.2. Study Strengths

The meticulous selection of the participants is a strength of this study. Despite the large fluctuations in the plasma concentration of BDNF in the human population, the investigation managed to create favorable conditions for observation by selecting three groups with no differences in the initial plasma concentration of neurotrophins. Another strength is the meticulous control during exercise. Each participant was individually controlled for body posture on a vibrating platform. Additionally, the introduction of the control of transmitting the vibrational stimulus to the head and neck was critical. The 12 weeks of training protocol allowed us to observe the body’s adaptation of the selected cohort group.

### 4.3. Research Perspectives

Future studies should focus on the heterogeneity of the samples including subjects for whom physical exercise activity is contraindicating or challenging to execute. It would be invigorating to look at how people with a reduced baseline BDNF level (as a result of psychiatric or neurological disorders) react to training with the inclusion of a vibrating platform. The presented observation indicates that the proposed training module is well-tolerated and not overly stressed, which allows it to be offered to people with health loads. 

## 5. Conclusions

The proposed, supervised form of effort on a vibrating platform is well-accepted and not overly stressed. Nevertheless, it cannot be a factor in enhancing neuroplasticity.

Neither a single training session nor a 12-week cycle of exercises on a vibrating platform causes changes in the concentrations of BDNF and cortisol in the serum of young, healthy women. The lack of changes in the cortisol level allows us to conclude that the stimulus applied was not stressful for the subjects. The necessary condition, however, seems to be the continuous control of the body posture on the vibrating platform. Comprehensive, cross-sectional, longitudinal research is needed to elucidate and better understand the underlying mechanisms and pathways of interaction engaging WBV and its physiological outcome. 

## Figures and Tables

**Table 1:** Criteria for exclusion from the study [1,29].

pregnancy, inflammation, infection, acute rheumatoid arthritis, arthrosis, epilepsy, pacemaker, metal and synthetic implants, blood clotting disorders, gallstones, kidney stones, severe diabetes, cardiac arrhythmia, and valve defects, coronary heart disease, back pain due to discopathy, hernia of the nucleus pulposus or spondylosis, tumors, severe migraines, mild positional dizziness; use of hormonal contraception; diagnosis of polycystic ovary syndrome or anovulatory cycles; special and elimination diets up to 3 months before joining the project; diagnosed or past psychiatric and endocrine diseases; high and moderate levels of physical activity.

**Table 2:** Age and basic anthropometric characteristics of the studied women.

	EG	AC	CG
Age [years]	21.65 ± 1.8	20.17 ± 1.75	19.53 ± 0.72
	*p* > 0.05		
Height [cm]	162.76 ± 7.51	164.67 ± 5.94	167.24 ± 4.56
	*p* > 0.05		
Weight [kg]	56.57 ± 7.18	59.43 ± 6.04	63.29 ± 8.71
	*p* = 0.029; post hoc: EG vs. CG		
BMI	21.31 ± 1.87	22.02 ± 2.91	22.57 ± 2.44
	*p* > 0.05		
PBF [%]	23.04 ± 6.11	25.62 ± 4.14	26.25 ± 5.76
	*p* > 0.05		
FM [kg]	13.34 ± 4.54	15.4 ± 3.85	17.03 ± 5.89
	*p* > 0.05		
FFM [kg]	43.24 ± 3.81	44.03 ± 2.84	46.25 ± 3.24
	*p* = 0.013; post hoc: EG vs. CG		

EG—experimental group; AC—active control group; CG—passive control group; BMI—body mass index; PBF—percent body fat; FM—fat mass; FFM—fat free mass.

**Table 3:** Plasma cortisol concentration in women from the vibration platform training group (EG), those exercising without additional vibration stimulus (AC), and the control group (CG).

Cortisol							
		I	II	III	IV	*p* inside the Group	*p* between Groups
EG	mean	261.4	215.8	242.1	210.6	0.166	I 0.618 II 0.099 III 0.767 IV 0.250
	SD	109.2	95.6	138.3	133.3		
	ME	244.1	228.8	186.9	147.9		
	MIN	132.2	91.31	130.4	104.1		
	MAX	493.5	376.2	523.2	496.1		
AC	mean	245.6	303.9	269.5	275.9	0.675	
	SD	125.7	168.9	133.2	154.7		
	ME	205.95	279.8	228.85	230.95		
	MIN	117.3	90.13	139.4	103		
	MAX	498.7	582.1	588.9	520.4		
CG	mean	206		277.4		0.067	
	SD	145.2		125.6			
	ME	170.4		246.7			
	MIN	62		148.3			
	MAX	621.6		537.7			

I—measurement made before the first training session, II—measurement made after the first training session, III—measurement made before the last training session, IV—measurement made after the last training session. SD—standard deviation; ME—median; MIN—minimum value; MAX—maximum.

**Table 4:** Plasma BDNF concentration in women from the vibration platform training group (EG), those exercising without additional vibration stimulus (AC), and the control group (CG).

BDNF							
		I	II	III	IV	*p* inside the Group	*p* between Groups
EG	mean	2.1	2.0	2.0	2.0	0.126	I 0.559 II 0.090 III 0.681 IV 0.124
	SD	0.9	0.8	0.8	0.7		
	ME	1.9	2.0	1.8	1.8		
	MIN	1.2	1.0	1.0	1.1		
	MAX	4.0	3.3	3.5	3.1		
AC	mean	2.6	2.5	2.3	2.4	0.315	
	SD	0.9	0.8	0.7	0.7		
	ME	2.4	2.4	2.1	2.2		
	MIN	1.5	1.6	1.4	1.6		
	MAX	3.7	4.1	3.5	3.8		
CG	mean	2.3		2.4		0.727	
	SD	1.3		1.6			
	ME	1.9		1.9			
	MIN	1.4		1.0			
	MAX	5.9		6.9			

I—measurement made before the first training session, II—measurement made after the first training session, III—measurement made before the last training session, IV—measurement made after the last training session. SD—standard deviation; ME—median; MIN—minimum value; MAX—maximum.

## Data Availability

The data presented in this study are available on request from the corresponding author.
